# Planning for and Assessing Rigor in Rapid Qualitative Analysis (PARRQA): a consensus-based framework for designing, conducting, and reporting

**DOI:** 10.1186/s13012-024-01397-1

**Published:** 2024-10-11

**Authors:** Christine P. Kowalski, Andrea L. Nevedal, Erin P. Finley, Jessica P. Young, Allison A. Lewinski, Amanda M. Midboe, Alison B. Hamilton

**Affiliations:** 1 Center for Clinical Management Research, VA Ann Arbor Healthcare System, 2215 Fuller Rd, Ann Arbor, MI 48105 USA; 2 VA Health Systems Research Center for the Study of Healthcare Innovation, Implementation, and Policy, VA Greater Los Angeles Healthcare System, 16111 Plummer Street, Sepulveda, CA, 91343 USA; 3 Departments of Medicine and Psychiatry and Behavioral Sciences, Long School of Medicine, UT Health San Antonio, 7703 Floyd Curl Drive, MC 7792, San Antonio, TX 78229 USA; 4 Seattle-Denver Center of Innovation (COIN) for Veteran-Centered Value-Driven Care, Seattle, WA USA; 5 Durham Center of Innovation to Accelerate Discovery and Practice Transformation, Durham Veterans Affairs Health Care System, 508 Fulton St, Durham, NC 27705 USA; 6 School of Nursing, Duke University, 307 Trent Drive, Box 3322 DUMC, Durham, NC 27710 USA; 7 Center for Innovation to Implementation (Ci2i), Palo Alto Health Care System, 795 Willow Rd, Menlo Park, CA USA; 8 Division of Health Policy and Management, University of California Davis—School of Medicine, Davis, CA USA; 9 Department of Psychiatry and Biobehavioral Sciences, David Geffen School of Medicine, University of California Los Angeles, Los Angeles, CA USA

**Keywords:** Rapid qualitative analysis, Rapid qualitative methods, Rigor, Validity, Rapid qualitative studies, Qualitative methods

## Abstract

**Background:**

The use of rapid qualitative methods has increased substantially over the past decade in quality improvement and health services research. These methods have gained traction in implementation research and practice, wherein real-time adjustments are often made to optimize processes and outcomes. This brisk increase begs the questions: what does rigor entail in projects that use rapid qualitative analysis (RQA)? How do we define a pragmatic framework to help research teams design and conduct rigorous and valid rapid qualitative projects? How can authors articulate rigor in their methods descriptions? Lastly, how can reviewers evaluate the rigor of rapid qualitative projects?.

**Methods:**

A team of seven interdisciplinary qualitative methods experts developed a framework for ensuring rigor and validity in RQA and methods suitable for this analytic approach. We conducted a qualitative evidence synthesis to identify gaps in the literature and then drew upon literature, standard procedures within our teams, and a repository of rapid qualitative training materials to create a planning and reporting framework. We iteratively refined this framework through 11 group working meetings (60-90 minutes each) over the course of one year and invited feedback on items to ensure their completeness, clarity, and comprehensibility.

**Results:**

The Planning for and Assessing Rigor in Rapid Qualitative Analysis (PARRQA) framework is organized progressively across phases from design to dissemination, as follows: 1) rigorous design (rationale and staffing), 2) semi-structured data collection (pilot and planning), 3) RQA: summary template development (accuracy and calibration), 4) RQA: matrix analysis (matrices), and 5) rapid qualitative data synthesis. Eighteen recommendations across these sections specify best practices for rigor and validity.

**Conclusions:**

Rapid qualitative methods play a central role in implementation evaluations, with the potential to yield prompt information and insights about context, processes, and relationships. However, guidance on how to assess rigor is nascent. The PARRQA framework enhances the literature by offering criteria to ensure appropriate planning for and assessment of rigor in projects that involve RQA. This framework provides a consensus-based resource to support high-level qualitative methodological rigor in implementation science.

Contributions to the literature

- Rapid qualitative methods can be a valuable tool for generating findings that support implementation activities such as assessing context, tailoring implementation strategies, and adapting interventions.
- Rapid qualitative analysis (RQA) is increasingly used in implementation efforts and reported in hundreds of publications, yet no planning and reporting framework currently exists to support or assess rigor in rapid qualitative methodology.
- The proposed framework fills a gap in the literature by offering criteria to ensure rigorous planning, conduct, and evaluation of projects involving RQA. It provides a consensus-based resource to support high-level qualitative methodological rigor in the application of rapid qualitative methods in implementation science.

## Background

Learning healthcare systems use various research methods to identify, assess, and improve quality issues, and support system-wide implementation and spread of evidence-based practices and successful innovations [1]. Qualitative methods have gained traction in implementation research and practice, wherein real-time adjustments are needed to optimize processes and outcomes and provide actionable results for real-time application. These efforts can require, for example, rapid assessment of context and innovation compatibility, timely tailoring of implementation strategies, swift identification of key constituents involved in organizational change, and multilevel assessment of intervention acceptability and potential need for adaptation [2]. All of these efforts can be informed by rapid turn-around of qualitative findings.

Traditional qualitative methods typically involve more time-intensive, in-depth data collection and analysis, including spending extensive time in the field and on analyzing data. In contrast, rapid qualitative analysis (RQA)—and study designs that utilize it—are purposely streamlined, using targeted, actionable, and feasible semi-structured data collection methods and corresponding analytic tools within abbreviated time-frames, without compromising rigor [3–6].

RQA and associated methods have been developed and refined over the past decade and are often utilized across quality improvement initiatives and health services and implementation research, with results and comparative work reported in numerous publications. For example, Nevedal et al. compared traditional versus rapid Consolidated Framework for Implementation Research (CFIR)-informed qualitative analysis and found RQA yielded similar results while taking less time, achieving project objectives, and maintaining rigor [5]. Other comparative work has yielded similar favorable assessments [7]. As rapid qualitative methods have made their way into a broad array of studies, funding proposals, and published results, key questions have emerged, including: what does rigor entail in projects drawing on RQA? How can authors articulate rigor in their methods descriptions? Lastly, how can reviewers of grant proposals and manuscripts evaluate the rigor of rapid qualitative projects (i.e., projects that include RQA)?

In 2022, recognizing the growing demand among VA researchers for training in RQA, Veterans Health Administration (VA) Quality Enhancement Research Initiative (QUERI) leadership encouraged the authors to apply for a QUERI Learning Hub (https://www.queri.research.va.gov/training_hubs/default.cfm), a mechanism oriented toward skill-building in the context of healthcare quality improvement. The “Rapid Qualitative Methods for Implementation Practice Learning Hub” (hereafter, Rapid Hub) was funded, with faculty spanning five VA healthcare systems and four universities. All Rapid Hub faculty are qualitative methods experts with advanced degrees in anthropology, gerontology, social work, nursing, psychology, epidemiology, public health, and behavioral science. All have extensive experience leading qualitative work and research teams in VA and in using RQA. Authors have conducted extensive rapid qualitative work within and outside of the VA, within and outside of the US, and within and outside of healthcare systems. A core goal of the hub was to develop a planning and reporting framework to enhance rigor and validity throughout all phases of projects that involve RQA, as well as to aid in evaluating the rigor of proposed or completed rapid studies.

### Extant qualitative checklists and criteria

Extant published criteria and checklists for qualitative research are designed to support traditional qualitative methods. For example, the Consolidated Criteria for Reporting Qualitative Research (COREQ, published in 2007) authors compiled 76 items from across 22 prior checklists to form criteria in three domains: 1) research team and reflexivity, 2) study design, and 3) data analysis and reporting [9]. The Standards for Reporting Qualitative Research (SRQR), published in 2014, defines standards for reporting qualitative research while preserving flexibility for various approaches and methods, and includes 21 items authors consider “essential” for complete transparency in reporting of qualitative research [10]. More recently, the Journal Article Reporting Standards for Qualitative Primary, Qualitative Meta-Analytic, and Mixed Methods Research in Psychology (JARS-QUAL) offers qualitative standards for psychology that “should be included in a research report to enable and facilitate the review process” [11].

There are limitations and strengths associated with using checklists to support conduct and reporting of qualitative research, summarized in Table 1. Critiques of qualitative checklists include the potential for inflexibility if too rigidly applied in the editorial and peer review process, and the implied expectation that all items on a checklist should be addressed. Inappropriate or misinformed critiques of qualitative work have been noted in the literature [12]. However, checklists can be a valuable tool for supporting more rigorous conduct and reporting of qualitative research, for example by ensuring methodological transparency [10]. Where checklists are flexible and appropriately applied, they can support editors and reviewers—who may have varying levels of training and experience in qualitative methodology—in more fairly and consistently appraising the merit/rigor of qualitative studies for funding or publication [11]. Transparency in qualitative methods description is important because it makes the assumptions and decisions accessible to readers and reviewers [10], and to the extent that qualitative checklists help with this, they are beneficial [10].

**Table 1:** Strengths and limitations of qualitative methods reporting checklists

Strengths	Limitations
Can assist researchers in planning and conducting qualitative studies [10]	Can be applied too literally, ignoring recommendations to approach their use flexibly [10, 11, 13]
Can help reviewers identify important information or considerations missing from study planning and reporting [11]	Components may not align across different checklists [11]
Can enable editors and reviewers to consistently and effectively appraise merit/rigor of qualitative work for funding or publication [14]	Can be inappropriately applied by those with limited expertise, potentially contributing to erroneous critiques where conformity to the checklist is not conferring rigor [12, 14]
Can support consistent and transparent reporting in qualitative work [10, 11, 13, 14]	Can impose elements that may not be true to the work that was conducted, e.g., misapplication of criteria and techniques from one qualitative tradition to another.[10–14]
Can provide clear guidance for reporting qualitative research, ensuring transparency [10, 11]	Are often used only at the end of a project, for reporting rather than to support rigorous planning and conduct throughout, [9] and may conflate rigorous reporting with rigorous study conduct [13]
Well-suited for traditional qualitative studies and analytic approaches	Not tailored or well-suited for use with rapid qualitative methods

Checklists or other evaluative criteria should be consistent with the qualitative approach of the work under review [11]. Despite increased interest in and utilization of RQA, no overarching framework to guide use of this approach has been published. Our objective is to share a consensus-based framework developed for projects using RQA, including appropriately designing and conducting projects, reporting on methods, disseminating results, and evaluating methodological rigor.

### Setting

The Rapid Hub provides national trainings and capacity-building for rapid qualitative methods within and beyond the VA research community. Our first effort as a Hub was to conduct a VA-based training needs assessment, which found that almost 100% of respondents (n = 194) wanted to learn more about establishing rigor in rapid qualitative projects. Based on this finding, we developed a framework to support learners.

## Methods

The Planning for and Assessing Rigor in Rapid Qualitative Analysis (PARRQA) planning and reporting framework was informed by the following methods: 1) a qualitative evidence synthesis to identify gaps in the literature [15], 2) Rapid Hub faculty meetings, and 3) a review of our team’s repository of rapid qualitative training materials.

First, we completed a qualitative evidence synthesis by searching PubMed and Google Scholar using the key terms: “qualitative standards,” “qualitative checklists,” and “qualitative rigor.” We did not find any checklists or tools designed specifically to help assess rigor in rapid qualitative projects. Over the course of two two-hour meetings, authors then developed the initial list of proposed planning and reporting guidance using the evidence synthesis and compiled training materials. Initially, all items generated by the authors were included. Next, over a one-year period, three authors (CPK, AN, EF) had 11 meetings (each ranging from 60–90 min) to develop and iterate the initial table to create the PARRQA framework. During those meetings, authors reviewed the most frequently asked questions from our Rapid Hub training sessions and mentoring calls and used this information to further refine the domains. After drafting the framework, the senior author (AH) refined it and then the manuscript was distributed to all authors. All authors, using this consensus-based approach, reviewed the draft PARRQA framework to ensure completeness, clarity, and comprehensibility. This continued in an iterative process of revisions over the course of a year, with additional refinement based on feedback from a December 2023 conference presentation and peer review of the manuscript.

We finalized the PARRQA framework with the goal of supporting planning and evaluating of 1) study designs and data collection approaches that are appropriate for rapid qualitative projects, 2) conduct of rapid qualitative projects, and 3) reporting and reviews of rapid qualitative projects. Given critiques of qualitative checklists, we designed the framework to also inform study planning. Therefore, this is a design, reporting, and methods tool, intended to provide useful, flexible guidance to study teams.

## Results

Table 2 presents the PARRQA framework and considerations to support rigor and validity of a rapid qualitative project throughout all phases. The framework intentionally focuses on semi-structured qualitative data collection methods, as RQA is designed for these methods (i.e., not for unstructured qualitative data collection methods).

**Table 2:** Planning for and Assessing Rigor in Rapid Qualitative Analysis (PARRQA): consensus-based framework for designing, conducting and reporting

**Item number**		**Guidelines to support rigor and validity**	**Important considerations**
1)	**Rigorous Design**	**Articulate the research question and finite purpose of the project**	Documenting the overarching research question will guide the entire project from designing the data collection instrument(s) through matrix development and reporting. Analysis timelines can be supported by identifying the finite purpose or deliverable. Furthermore, it is helpful to specify the immediate purpose of the project
2)		**Describe the rationale for using rapid qualitative methods**	Potential rationales for using a rapid qualitative approach include: • Project requires rapid turnaround of findings • Need to work within constrained resources and timelines • Project has a narrow scope or focused research question that can be addressed using semi-structured (and perhaps some structured) qualitative data collection • Sample has high information power[19] (See additional examples in manuscript text)
3)		**Define what is meant by “rapid qualitative analysis.”**	Describe the approach, including data types (e.g., transcripts, audio files, notes), collection, analysis, and dissemination, with appropriate citations (i.e., of the rapid technique being employed) • Should include a more explicit description of the steps taken in preparing the data collection instrument(s), collecting data, summarizing data from each interview, developing a matrix, and so forth
4)		**Consider whether a theory, model, or framework will be used to inform the study and if so, why and how**	Using theories, models, and frameworks can be helpful in focusing the scope and content of a rapid qualitative project but are not required. If using one: • Document the rationale for use • Define which constructs will and will not be included and why • Explain how constructs will be integrated into data collection and analysis (e.g., reflected in the interview guide, structured summary, and matrix)
5)		**Define the intended timeframe of data collection, analysis, and products/deliverables**	Defining the intended timeframe will aid in monitoring progress over the course of the project: • Intended time frame should include the time for planning, collecting data, analyzing, generating products, and disseminating results • In longitudinal studies, it may be necessary to specify time frames for each study phase (e.g., preparation, implementation, and sustainment)
6)		**Plan for appropriate staffing**	Planning for staffing should ensure: • Sufficient dedicated staff time (FTE) • Account for (a) number of and length of interviews; (b) time needed for taking notes and calendar blocking; (c) time needed for data management and cleaning; (d) time needed for analysis; (e) time needed for write-up/dissemination • Optimally interviewers should be involved in both data collection and analysis
7)		**Explain the purpose and timeline of the study, communication plan, and roles to all team members**	• Clarifying roles supports more efficient and rigorous collaboration • Efficiency will be increased by ensuring that each team member has a clear understanding of their role(s) and responsibilities • Consistent team communication can prevent drift in data collection or analysis approach among team members
8)	**Data Collection**	**Develop and refine data collection tools to address specified research questions**	• Using semi-structured data collection instruments that closely align with the research question and theory/model/ framework (if relevant) is advised for rapid qualitive projects • Interview guide questions should generally be more focused and targeted, but should remain open-ended • Interview length should be kept low burden for participants (e.g., 30–45 min) • Any other data collection tools, e.g., structured templates for notetaking or observations, should be similarly focused
9)		**Pilot data collection instruments to ensure they are clear, feasible, and appropriately targeted**	• Collecting data consistently helps produce more rigorous and valid findings for rapid qualitative approaches • Ensuring that semi-structured interview or focus group questions are comprehensible and clear to participants is critical prior to beginning formal data collection • Ensuring that observational templates are understood by observers and are easy to use consistently
10)		**Develop a plan for review throughout the data collection process**	• Consider regular review to ensure that data collection is consistent and adequate • Review can include weekly de-briefs, interview reflections, and methods feedback sessions
11)	**RQA: Summary Template Development**	**Develop and pilot test a user-friendly summary template**	• Develop a clear and systematic plan for summarizing data, whether distilled from notes, audio recordings, or transcripts • Key summary template domains are typically and at least initially based on the questions from the interview guide • The template should be focused (approximately 2–3 pages)
12)		**Ensure there are cross-references to raw data to support continuous comparison and validation necessary for RQA**	• Determine if data will be distilled from notes or a transcript, and whether using a summary template or summarizing directly into a matrix • Transcript line numbers, brief quotes, and time stamps can be used to link to the raw data, allowing analysts to re-examine the original data as needed for clarification, validation, and expansion
13)		**Develop summaries that are accurate and concise, but detailed enough to meet project aims**	Developing a good, structured summary includes an emphasis on being: • Connected to and reflective of the raw data • Brief yet thorough enough to stand alone • Minimally interpretive • Streamlined but complete Discuss whether reflective writing will be done during the summary phase or other analysis time point and where that reflective writing will be placed
14)		**Identify training and calibration processes to ensure consistency and accuracy in summaries**	Strategies may include: • Prioritize information that is relevant to the research question • Ensure consistent approach to formatting, quotations, timestamps/ line numbers, and general amount of detail (appropriate length) • Discuss the importance of accurate description vs. interpretation when summarizing data • Use a training dataset for several researchers to establish consistency before dividing up the work • Meet regularly with the team during analysis to discuss / resolve discrepancies • Use a secondary reviewer to spot check/audit the summaries [21] • Employ consistent team processes for data condensation throughout the project
15)	**RQA: Matrix Analysis**	**Plan qualitative matrix structure to reflect project aims/ questions**	• More deductive analytic approaches (i.e., a priori) are preferable given rapid timelines • Matrices are often developed prior to data collection and should map directly to the interview questions and/or domains • Ensure domains are clearly defined and assess their appropriateness (and potential need for revision) throughout analysis
16)		**Describe use of software for matrix analysis (e.g., Excel, Word)**	• Microsoft Word and Excel are useful for matrix analysis • Qualitative data analysis software may not be needed, and its non-use should not signify lack of rigor
17)		**Develop a plan for review throughout matrix analysis**	• A senior person (lead analyst and/or investigator) should complete early review of matrix data input for each analyst (e.g., level of detail and relevance to the research question/s) • Some periodic monitoring and discussion should continue throughout the analysis
18)	**Rapid Qualitative Data Synthesis**	**Conduct synthesis that is rigorous and responsive to research priorities**	• Be specific about what your synthesis process will entail and how it aligns with the analysis conducted o Description can be used to develop a higher-level summary o Describe patterns within and/or across cases/domains o May also include more interpretation to relate findings to existing literature or interested parties • Synthesis can be dependent on your dissemination plan • Engage in data review to ensure accuracy and completeness of key points through cross checking and cross comparison

### Rigorous design

#### Articulate the research question(s) and finite purpose of the project

In implementation and health services research, most core elements of study design emerge from addressing the project’s central research or evaluation question(s). This is also true for projects using rapid qualitative analysis. During initial project planning, it is important to articulate and document the research question(s) guiding qualitative data collection and analysis, from designing data collection instrument(s) through development of summary templates and matrices, and reporting. Furthermore, it is helpful to specify time-sensitive project goals, e.g., a product needs to be generated within a specified timeline, including executive summaries, brief presentations, synthesized reports for team members and/or implementation partners. This specification provides appropriate focus and scope for the rapid qualitative project but does not preclude the team from generating additional products, perhaps outside of the immediate timeline. The research question should be revisited to ensure its relevance and to document any new questions that arise during data collection and analysis.

#### Describe the rationale for using RQA

**Fig. 1:**
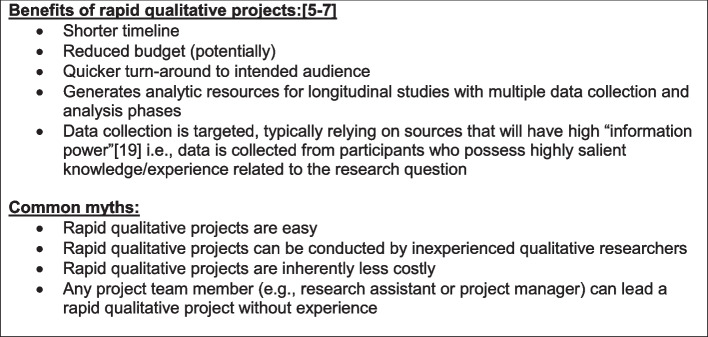
Benefits and common myths about rapid qualitative projects

When designing a study, teams should describe and document the rationale for using RQA (see Fig. 1). Projects typically use rapid analysis when a project requires rapid turnaround of findings, e.g., in an implementation project where context and barriers are being assessed and information is shared with local sites or implementation teams in a timely way. Rapid methods are best suited to projects that have a narrow scope or focused research question (see #1). Additional examples for when it is appropriate to use RQA include when time is limited and there is urgent need to deliver findings on schedule, as in a pilot or other brief study; in phased work where next steps are data-dependent; when operational partners or policymakers are in need of mission-critical data; or when conducting longitudinal work with multiple data collection waves [5]. RQA is also appropriate when the qualitative component is not the focus (e.g., in some types of mixed methods designs), in generating qualitative findings to explain unexpected quantitative findings, and in developing high-level takeaways for dissemination via publications or other types of products for partners and other constituents.

In considering the rationale(s), it is important to dispel common myths about RQA (see Fig. 1)—first and foremost, that this approach is easier and can be done by those with little or no qualitative experience. This is not an appropriate nor accurate rationale. On the contrary, shorter timeframes often require greater focus, intention, and cognitive load during both data collection and analysis, and benefit from methods leadership with specific expertise/training in this approach; as with all methods, the resulting rigor (or lack thereof) derives from the expertise of the researchers and the care taken to align with research best practices throughout the process [5].

#### Define what is meant by “rapid qualitative analysis”

Teams should describe their approach to using RQA, including the methods (e.g., semi-structured interviews), data types (e.g., transcripts, audio files, notes), data collection instruments, analysis plans, and intended or priority products. Regarding analysis, researchers should provide detailed information about how methods aligned with the analytic approach, including how data collection instruments were prepared and used, how summary templates and matrices were designed, when, and by whom, and so forth. This offers a transparent and replicable methods account to reviewers and readers. Given the variety of qualitative methods and approaches available, citations should be provided and specific to the approach used.

#### Consider whether a theory, model, or framework will be used to inform the study and if so, why and how

In designing projects that will use RQA, it is important to consider whether theories, models, and/or frameworks will be used, and if so, in what ways. Models, theories, and frameworks can be used, but are not required, in rapid qualitative projects [17]. Deciding if one should be used, and which to apply, is dependent on the type of research (e.g., this would be expected in implementation research), the project goals, and the research question(s). If planning to use a theory, model, or framework, it is important to document the rationale behind the decision, which core constructs and elements will be included, how they will be integrated as part of data collection (e.g., reflected in the interview guide) and analysis, and if adaptations will be needed to better align with RQA. For example, frameworks such as the Consolidated Framework for Implementation Research (CFIR) can be adapted such that only necessary and focused portions are utilized for data collection and analysis, i.e., by including only constructs expected to be relevant to the focused research question [5].

#### Define the intended timeframe of data collection, analysis, and products/deliverables

To achieve rigor throughout the rapid qualitative project, the expected timeline for methods should be delineated from the beginning of the study, with regular check-ins to ensure the project remains on schedule. In addition, researchers should state when rapid analysis occurs in relation to data collection. Beyond facing delay, projects that do not adhere to planned timelines may not achieve expected sample sizes or allow adequate time for planned analyses, negatively impacting study rigor.

#### Plan for appropriate staffing

Ensuring the study design is feasible is important for projects that involve rapid turnaround of qualitative findings, given the abbreviated timeline. Feasibility and timeline are tied to staffing, which relates to budgeting. Qualitative data collection geared toward RQA should be conducted by a team of at least two for greater rigor and staff should have sufficient dedicated time; the number of team members is proportional to the scope of the study (e.g., number of sites, number of participants, volume of data collection). Ideally interviewers are involved in both data collection and analysis, but it is also possible that some staff will be more responsible for data collection, while others focus on analysis.

Projects involving RQA are not necessarily inexpensive. For example, a rapid project may require several experienced team members working intensively to generate the intended product. When constructing a project budget, the intended timeframe of data collection and analysis; feasibility of recruitment; number, type(s), and length of data collection approaches; data management and cleaning; and analysis and write-up should all be considered in determining staffing needs. Without sufficient staff, rigor will be difficult to achieve.

#### Explain the purpose and timeline of the study, communication plan, and roles to all team members

In projects with rapid turn-around timelines (as in all projects), it is critical for team members to understand the purpose of the study, as well as the timeline, expectations, and deliverables in relation to the timeline (e.g., a certain number of interviews completed by a certain date). Efficiency will be increased by ensuring team members understand their role(s) and responsibilities; clear roles will support efficient and rigorous collaboration. Teamwork is essential to rapid qualitative projects because of the intensive timelines and need for consistent data collection and analysis across the team. Effective and regular team communication can ensure consistency and rigor and contribute to a favorable work environment and timely completion of deliverables.

### Data collection

#### Develop and refine semi-structured data collection instruments to address specified research questions

Projects involving RQA rely on semi-structured data collection instruments (e.g., interview guides, observation templates). This means specifying the focused, yet still open-ended questions and the sampling frame. While traditional qualitative data collection tools may cover a breadth of topics, domains, and experiences and may be more exploratory, we recommend designing rapid data collection with focused questions (hence, less exploratory) designed for specific samples/settings—think of this as asking THESE people to answer THESE questions [19]. The qualitative data collection instruments should be designed by team members with qualitative methods expertise, with input from other team members. Semi-structured interview guide questions should be inviting, accessible, and analyzable, and the number of questions should be geared toward the time available for the interview [4]. In rapid qualitative projects, work should be done prior to data collection to facilitate rapid analysis, e.g., the interview guide questions can be mapped to key topics (and framework constructs, if relevant) in advance, with the caveat that these topics will need to be revisited during and after data collection to ensure that they are still relevant and to explore unanticipated topics. Teams should map the interview questions to the rapid analysis matrix during early planning. This is in contrast to traditional qualitative projects where analytic work may be done after data collection. The guide or other data collection instrument should be reviewed to ensure its feasibility, relevance, and productivity. The qualitative methodologist and/or analyst should review the guide with the study’s team to ensure that unnecessary questions are not included, the number of questions is feasible for the time allotted for the interview, and that questions effectively address the key aims.

#### Pilot data collection instruments to ensure they are clear, feasible, and appropriately targeted

We recommend pilot testing data collection instruments with appropriate testers (e.g., patients, clinicians) since rapid qualitative projects require focused and sometimes brief opportunities for data collection. Pilot testing and subsequent revision contribute to more rigorous and valid findings, e.g., by ensuring that interview questions are accessible and relevant, clarifying terminology that is unclear to interviewees, understanding how interviewees may respond to questions, determining whether the interview is too detailed/lengthy, and assessing whether the guide is addressing the research question(s). Semi-structured observational templates also need to be pilot tested among the team-members who will be conducting observations to ensure feasibility, relevance, and consistency.

#### Develop and use a plan for review throughout the data collection process

Consider regular data review that is documented and adhered to throughout data collection. This includes weekly de-briefs, interview/fieldwork reflections, and quality checks by methods experts. These steps to support rigor and validity can prevent drift and discrepancies in the data collected. This is important for rapid qualitative data collection because it occurs over a shortened timeline and therefore, requires early adaptions and refinements to ensure data collected is focused and addresses the research question(s).

### RQA: summary template development

#### Develop and pilot test a user-friendly summary template

Develop a clear and systematic plan for summarizing data, whether distilled from notes, audio recordings, or transcripts. As opposed to traditional qualitative methods where codes may not be developed until the onset of analysis, key summary template domains are initially based on domains in the data collection instruments (see #8). Therefore, the summary template can be drafted prior to data collection. The summary template should be user friendly and not unwieldy, and it should be reviewed by the research team to ensure that it covers all the topics in the data collection instruments. The team will need to consider whether multiple summary templates are needed in one project, depending on the nature of the sampling frame and potential variations across data collection instruments (e.g., different interview guides for patients versus providers).

#### Ensure there are cross-references to raw data to support continuous comparison and validation necessary for rapid analysis

Condensing data occurs quickly in RQA. Therefore, it is important to maintain connections to the raw data to support continuous checking and emergent interpretations (e.g., identifying novel results or recommendations). Throughout the summarization process, we recommend including references to raw data. Given that the summaries are designed to be concise encapsulations of data collection episodes, this is a way to enable quick access to important quotes or areas of narrative for later reporting. For example, if the team is creating summaries from transcripts, transcript line numbers can be listed alongside bulleted summary points, allowing analysts to return to the transcript and re-examine the original data as needed for clarification and validation. If notes or audio files are being used instead of transcripts, providing time stamps during interview note-taking or while listening to the recordings can be used to link summaries to relevant segments of the audio files. Transcription may or may not be used. Sometimes it is necessary due to time/budget constraints to generate a summary from notes and supplement with the audio file when needed. Teams will need to practice cross-referencing to whatever form(s) of data are being used for analysis. We recommend creating a training dataset (e.g., 2–3 transcripts) and examining (and potentially standardizing) the ways in which team members cross-reference the raw data. If possible, we encourage researchers to try to analyze data with and without transcripts with the training dataset to determine what works best for a particular project and team members.

#### Develop summaries that are accurate and concise, but detailed enough to meet project aims

We recommend that researchers write a concise summary immediately after data collection to provide an accessible and accurate window into that episode [20]. Summaries should average 2–3 pages. Good summaries are: 1) based on and explicitly connected to the raw data; 2) brief yet thorough enough to give an overall sense of the data collection episode; 3) minimally interpretive; and 4) accurate reflections of the data collection process. The team should discuss whether reflective writing will be done while preparing summaries or at other junctures during the analytic process, and where that reflective writing will be placed (e.g., in a separate document/memo, in comment bubbles or brackets within the summary). These decisions will vary by team and by project and should be documented.

We strongly recommend calendar blocking for writing summaries at the conclusion of data collection episodes, unless transcripts will be used to prepare summaries in which case summaries can be completed as transcripts are received [5]. Plan for 1–2 h to write each summary. When the summaries are created as soon as possible after data collection, it adds rigor because it builds on researcher recall. This also ensures that summaries are created in a timely manner to meet project deadlines and goals rather than creating a backlog of analytic work. Summaries written immediately after data collection should be cross-checked against audio files or transcripts as those files become available, to ensure accuracy.

#### Identify training and calibration processes to ensure consistency and accuracy in summaries

Training and calibration are necessary to support rigor in the completion of summaries. Initial summaries can vary greatly within teams, with some being too brief and others too detailed, or some being too interpretive and distal from the data itself. This is why the “test-driving” step of RQA is essential and should not be skipped, no matter how experienced the team [3]. This step involves having all team members review the same 2–3 data sources and prepare summaries independently, and then review and compare the summaries.

The lead qualitative methodologist should also periodically audit a cross-section of summaries (e.g., 20%, with a set from each person completing summaries) to ensure: 1) appropriate length of the summary; 2) alignment between the summary and the data collection domains; 3) consistent formatting, use of quotations, and amount of detail; 4) descriptive rather than interpretive summaries; and 5) description that can be adequately understood by the team such that they have a solid sense of what was heard or observed in the data collection episode [21].

Teams should ensure that summaries of semi-structured interview data are trustworthy, accurate representations of participants’ experiences and perspectives. For example, in one of our projects, the summary stated that the “patient was complaining.” However, a secondary analyst noted that in the transcript, the patient expressed “concerns” about her care. Extrapolating to “complaining” is a form of interpretation that is not appropriate forRQA; instead, we stay close to the data and use the participant’s words, even in snippets of phrases [20].

Of note, there are projects where solo RQA takes place by necessity, due to a lack of staff or funding, and there are steps that can be taken to ensure rigor, such as verification of summaries with audio files or transcripts. We recommend pausing, perhaps for a standardized time-period, between developing the initial summary and verifying the summary, to allow for reflection and learning.

### RQA: matrix analysis

#### Plan qualitative matrix structure to reflect project aims/questions

Plan your qualitative matrix structure to reflect project aims/research questions. Domain names from the summary template can be column headers in the matrix, which means that a preliminary matrix can be created prior to onset of data collection. To facilitate rigorous analysis of the matrix, the research team should continuously and systematically review each domain within and across subsamples (if relevant) and memo extensively on observations and potential themes to explore (see #18). Matrix analysis in rapid qualitative projects may not be as exploratory as in other types of qualitative projects given the focused nature of rapid turn-around work.

#### Describe use of software for matrix analysis

It is important for researchers to specify if and how software programs are used to support RQA [5]. Summaries are typically developed in MS Word or PowerPoint, and matrices in MS Word and/or Excel.[5] RQA was specifically designed *not* to necessitate specialized qualitative data analysis software (QDAS). However, if QDAS is used, its use needs to be thoroughly explained and justified. To this point, reviewers and editors should not assume use of QDAS inherently imparts rigor, not should it be assumed that the lack of QDAS signifies lack of rigor.

#### Develop a plan for review throughout matrix analysis

Data included in the matrix should be concise. Lengthy segments from the raw data should not be included, as the matrix will become unwieldy. As with the summarizing process, teamwork is critical to the rigor of matrix analysis. Some teams copy and paste their summaries into matrices, while others engage in data transformation as matrices are developed [23]. The lead qualitative methodologist should oversee the development of matrices to ensure their alignment with project goals and should review matrices for accuracy and consistency, including monitoring for consistent level of detail and whether the text entered is answering the research questions, well-organized, and focused [5]. All observations about the analysis process should be shared with the team in order to educate and ensure consistency across the team. Throughout matrix analysis, team members should document (e.g., in memos) their efforts to ensure rigor and have this information ready for reporting in a manuscript or other forms of dissemination.

### Rapid qualitative data synthesis

#### Conduct synthesis that is rigorous and responsive to research priorities

Researchers should outline their synthesis approach and how it addresses research questions. The synthesis process is enabled by the design of the summaries and matrices. Analysts can review a summary or a column in a matrix to compare across cases, whether that be a type of interviewee (physicians compared to nurses, for example), or sites, clinics, or units, for example. Synthesis details and steps will vary based on goals for dissemination. For example, if the data will be fed back to a site or operational partner, a synthesis output may make more sense as a PowerPoint presentation or brief report—something understood and digestible by the recipient. Whenever possible, we recommend visual displays and other ways of conveying details. If the goal of the synthesis is a manuscript and quotes will be utilized, the data linkages embedded in summaries (see #12) will help to find pertinent quotes to illustrate concepts. Engage in data review to ensure accuracy and completeness of key points through cross-checking and cross-comparison to enhance rigor and validity of synthesized findings. In synthesis and dissemination materials, we recommend specifying whether and how formative feedback (e.g., from participants, constituents) informed the analytic process.

### Rigor and validity in reporting and publishing on rapid qualitative projects

Journal expectations are not always consistent, and authors may be unsure of what to mention and describe about their rapid qualitative projects. We provide a suggested planning and reporting framework to aid reviewers and editors in assessing the rigor and validity of rapid qualitative projects in Table 3. Editors and reviewers can use Tables 2 and 3 to help evaluate the rigor of rapid qualitative projects. We also suggest that it may be helpful to have editors select reviewers with appropriate qualitative expertise for the methods under review. [11] Restrictive word limits may be problematic for describing study design and conduct in detail and can lead to authors leaving out information on methods that help readers to contextualize their results. If not possible within the manuscript, editors could consider publication of supplemental materials, such as inclusion of a completed Table 2 as part of the submission and publication process. A well-articulated research question and rationale for why and how RQA was used should be included in reporting to the journal. It is also useful to communicate to prospective reviewers that qualitative checklists are flexible tools to guide the authors/reviewers rather than universal requirements for all qualitative methods (see also Table 1). Reviewers should also check for rationale for why the authors are using RQA (see Table 2, item 2), review detail in the description of semi-structured qualitative methods (see Table 2, item 3), assess for indications of rigor in the methods (see Table 2, items 8–14, 17–18), and check for indications of teamwork in data collection and analysis (Table 2, items 7, 10, 14, 17).

**Table 3:** Guidelines to support rigor and validity in reporting and publishing rapid qualitative approaches

Item number	Suggestions for peer-reviewed journal reviewers and editors
	Determine and select appropriate reviewers with rapid qualitative methods expertise
	Allocate extra words when feasible to allow adequate descriptions of qualitative methods. If not possible, consider publication of supplementary materials
	Qualitative checklists are intended to be flexible tools rather than universal requirements for all qualitative methods (see Table 1)
	Check for rationale for why the authors are using rapid qualitative methods (see Table 2, item 2)
	Review detail in the description of rapid qualitative methods (see Table 2, item 3)
	Assess for indications of rigor in the methods (see Table 2, items 8–14, 17–18)
	Check for indications of teamwork (if applicable) in data collection and analysis (Table 2, items 7, 10, 14, 17)

It is important to note that in addition to grant and manuscript reviews, reviewers or operational partners receiving qualitative or visual display reports based on rapid qualitative analyses can also use Tables 2 and 3 to evaluate rigor.

## Discussion

Rapid qualitative methods are expanding in uptake and utilization. In contrast to traditional qualitative methods, rapid methodology can allow data to be collected and analyzed and findings disseminated quickly within a project or grant cycle. However, there has been a lack of clarity in the field about when RQA should be used and how it can be carried out rigorously.

The Planning for and Assessing Rigor in Rapid Qualitative Analysis (PARRQA) framework was developed to support rigor and validity in projects using RQA, including study design, conduct, write-up, and review. This consensus-based framework offers a checklist covering 18 key considerations related to: 1) rigorous design (seven elements), 2) data collection (three elements), 3) RQA: summary template development (four elements), 4) RQA: matrix analysis (three elements), and 5) rapid qualitative data synthesis (one element). This framework can support continuous improvement in the rigor and validity of RQA.

Journal editors and grant and journal reviewers can evaluate the methodological rigor and validity of rapid qualitative manuscripts and grants under review using the framework outlined in Tables 2 and 3. This framework can help reviewers to determine information that may be missing from methods sections and ask clarifying questions. Although word counts are limited, it is important to not only describe methods, but also describe if RQA training and ongoing monitoring is available.

Although rigor is discussed in some literature related to rapid qualitative projects, [5] there are no comprehensive recommendations consolidating elements that affect rigor and validity in projects using RQA. Although not discussed as guidelines, Smith and colleagues’ book on rapid evaluation highlights how rapid methods generally require careful management and oversight to support effective teamwork and ensure consistency of approach, and recommend that rapid qualitative teams be adequately resourced, and that teams should standardize processes or tools such as structured templates, build rapid evaluation skills and expertise, and ensure effective communication [24]. The PARRQA framework aligns with these general principles, while going further to operationalize and define specific recommendations to aid investigative teams in planning and reporting. A further advantage of the PARRQA framework is that suggested guidance is pragmatic and succinct, intended to make RQA accessible to a wider audience. This framework also provides greater detail by outlining recommendations to plan for management and standardization and allot for adequate staffing. Table 2 additionally provides considerations for when it is appropriate to utilize RQA, and ways to ensure rigor throughout planning, data collection, analysis, and reporting, including: piloting of data collection instruments, summary templates, and matrices; audits to ensure consistency across analysts; and ongoing training and supervision.

### Limitations

The PARRQA framework has not yet been formally applied in its entirety to a project or paper; the Hub faculty is currently working on developing examples of its application. This was also the case when other qualitative checklists were introduced [9–11]. Additionally, this planning and reporting framework was created based on literature review and developed and refined over a 12-month period by US-based qualitative methodologists, all of whom conduct research primarily in healthcare settings. We encourage other researchers/evaluators to use the PARRQA framework and provide feedback. We designed this framework broadly to work for a range of semi-structured data collection methods, particularly individual interviews, focus groups, and periodic reflections [25]. We have less experience using RQA with semi-structured observational data, but work in this area is ongoing. As previously noted, RQA is not appropriate and is not recommended for use with unstructured qualitative data [8]. There may be some adaptations required for specific types of methods, and this will be an important consideration for future work.

## Conclusion

RQA is a valuable tool in implementation evaluations, yielding critical in-depth information and insights about context, process, and relationships. However, guidance on assessing rigor in projects using RQA is nascent. The consensus-based PARRQA framework fills a gap in the literature by offering criteria to ensure rigorous planning, conduct, and evaluation of rapid qualitative projects. The PARRQA framework provides an expert-guided resource to support high-level methodological rigor in real-world qualitative implementation research.

## Data Availability

Not applicable.

## Abbreviations

PARRQA framework: Planning for and Assessing Rigor in Rapid Qualitative Analysis
CFIR: Consolidated Framework for Implementation Research
QUERI: Quality Enhancement Research Initiative
COREQ: Consolidated Criteria for Reporting Qualitative Research
SRQR: Standards for Reporting Qualitative Research
JARS-QUAL: The Journal Article Reporting Standards for Qualitative Primary, Qualitative Meta-Analytic, and Mixed Methods Research in Psychology
VHA: Veterans Health Administration
